# Association of composite dietary antioxidant index with incidence and mortality of aortic aneurysm and dissection: insights from the UK Biobank

**DOI:** 10.3389/fnut.2026.1713690

**Published:** 2026-02-13

**Authors:** Shengwei Lai, Handai Qin, Minghao Liu, Zhiwei Lai, Shuaifei Ji, Dan Rong, Wei Guo

**Affiliations:** 1 Department of Vascular Surgery, First Medical Centre of Chinese PLA General Hospital, Beijing, China; 2 Medical School of Chinese PLA, Beijing, China; 3 Tianjin University of Technology, Tianjin, China; 4 Department of Burn and Plastic Surgery, PLA Rocket Force Characteristic Medical Center, Beijing, China

**Keywords:** antioxidant, aortic aneurysm and dissection, composite dietary antioxidant index, oxidative stress, UK Biobank

## Abstract

**Background:**

The composite dietary antioxidant index (CDAI) is a scoring system designed to assess overall dietary antioxidant capacity and has been associated with a reduced risk of cardiovascular diseases. However, its specific impact on aortic aneurysm and dissection (AA/AD) remains unclear. This study aimed to investigate the associations of CDAI with both the incidence and mortality of AA/AD.

**Methods:**

In this UK Biobank-based study, univariate and multivariate logistic regression models were used to assess the association between CDAI and the incidence of AA/AD, the association of CDAI with mortality was evaluated using Cox proportional hazards models. We employed restricted cubic spline (RCS) analyses to examine potential linear or non-linear relationships between the key nutrient components of the CDAI and the outcomes. Furthermore, mediation analysis was performed to assess the potential mediating effects of selected metabolic indicators.

**Results:**

A total of 172,450 participants were included in this study, of whom 1,486 developed AA/AD. Univariate logistic regression analysis revealed a significant inverse association between CDAI and the incidence of AA/AD (OR = 0.93, 95% CI: 0.88–0.99, *p* = 0.024). A significantly lower risk of AA/AD mortality was observed in participants within the highest quartile of CDAI compared to those in the lowest quartile (HR = 0.83, 95% CI: 0.71–0.96, *p* = 0.018), based on the Cox regression analysis. RCS analysis indicated a linear relationship between CDAI and the mortality of AA/AD (P for overall < 0.001; P for nonlinear > 0.05). Furthermore, mediation analysis suggested that uric acid, neutrophil-to-lymphocyte ratio (NLR), C-reactive protein (CRP), and high-density lipoprotein cholesterol (HDL-C) mediated the association between CDAI and AA/AD incidence.

**Conclusion:**

This study supports the pathogenic role of oxidative stress and inflammation in AA/AD, demonstrating that a higher CDAI is associated with lower incidence and mortality of AA/AD in a UK-based adult population. These findings provide new insights, suggesting that dietary antioxidant intervention could serve as a potential preventive strategy against these conditions.

## 1 Introduction

AA and AD are cardiovascular conditions associated with high mortality rates. Acute aortic syndromes, particularly rupture, represent a leading cause of death. Since AA and AD are often asymptomatic until the occurrence of complications such as dissection or rupture, mortality rates for this condition can reach up to 50% (1). Aortic dissection typically initiates from an intimal tear, allowing blood to propagate distally through the medial layer of the aortic wall. The most severe outcome is aortic rupture, leading to fatal exsanguination. Although the incidence of AD and AA is relatively low, their case-fatality rate is substantial. Epidemiological data from Western countries indicate that the annual rupture rate reaches approximately 3 and 9 per 100,000 person-years for thoracic and abdominal aortic aneurysms, respectively, the incidence of acute aortic dissection is comparable (2). AA and AD share common environmental risk factors, including smoking, male gender, alcohol consumption, advanced age, and hypertension. Furthermore, emerging evidence suggests that dietary habits significantly influence cardiovascular risk. An imbalance in diet, marked by excessive sugar and trans-fats alongside insufficient vitamins and fiber, contributes to a higher risk of cardiovascular diseases (3, 4).

Oxidative stress is central to the pathogenesis of AA/AD. This condition is fundamentally characterized by a dysregulation of the equilibrium between reactive oxygen species (ROS) production and endogenous antioxidant defenses. The resultant imbalance triggers a cascade of pathogenic events, including vascular endothelial injury, apoptosis of smooth muscle cells, and breakdown of the extracellular matrix (5, 6). Furthermore, oxidative stress triggers an inflammatory response. The recruitment and infiltration of inflammatory cells exacerbate endothelial damage and promote the degradation of elastic fibers in the medial layer, thereby compromising the structural integrity of the aortic wall.

The CDAI serves as a comprehensive index designed to quantify the collective antioxidant capacity from various nutrients in the diet. It was first introduced by Wright et al. (7). This index quantifies the intake of six key antioxidants: vitamin A, vitamin C, vitamin E, zinc, selenium, and beta-carotene. By integrating these components, the CDAI overcomes the limitations of analyzing single nutrients and provides a holistic reflection of overall dietary antioxidant capacity. The vascular protective effects of dietary antioxidants are mediated through the scavenging of ROS, suppression of pro-inflammatory cytokine expression (e.g., TNF-α and IL-6), and regulation of key enzymes in vascular remodeling like matrix metalloproteinases (MMPs) (8). Adequate dietary antioxidant capacity may therefore protect against AA/AD by attenuating oxidative damage and stabilizing the aortic wall.

While numerous studies have analyzed the association between the antioxidant profile represented by the CDAI and various oxidative stress-related diseases, its specific impact on AA/AD remains unclear and lacks robust supporting data. Therefore, we conducted an analysis using comprehensive UK Biobank data to investigate the association between CDAI and both the incidence and mortality of AA/AD.

## 2 Methods

### 2.1 Data source and study participates

**Figure 1:**
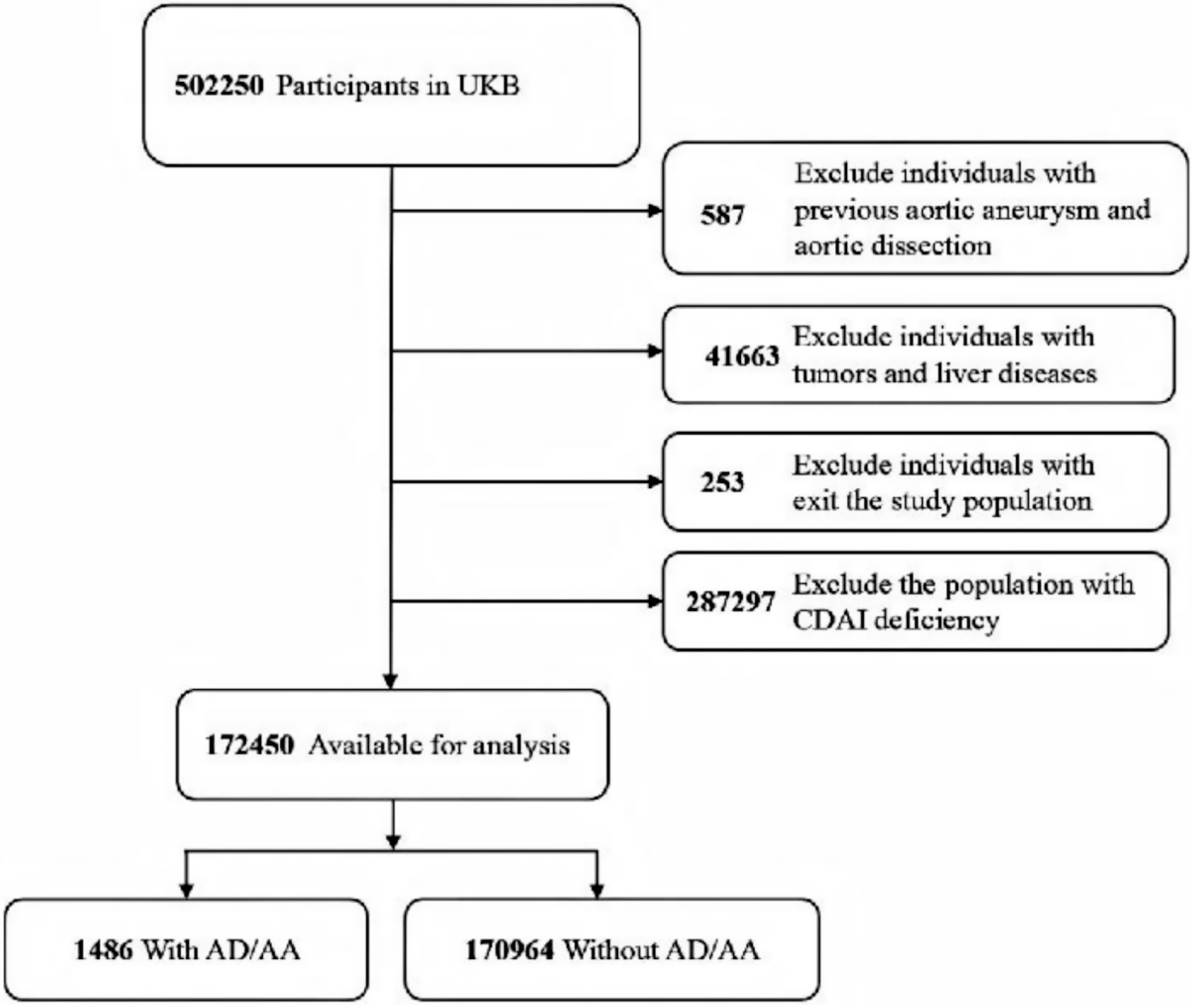
Flowchart of the study participants.

The UK Biobank is a major prospective cohort study established to support a broad range of biomedical research. The study systematically collected information on lifestyle, biological samples, and health data from approximately 500,000 adults aged 37–73 in the United Kingdom through questionnaires. It received approval from the Northwest Multicenter Research Ethics Committee (MREC) and all participants provided written informed consent. In this study, to avoid potential confounding effects of pre-existing conditions or their treatments on participants’ dietary patterns and biomarker levels, we excluded participants with a pre-existing diagnosis of AA/AD (*n* = 587), those with severe liver disease or malignant tumors (*n* = 41,663), individuals who withdrew from the study (*n* = 253), and those with missing or incomplete CDAI data (*n* = 287,297). Consequently, a total of 172,450 participants were ultimately included in the analysis. The enrollment of participants for this study is described in Figure 1.

### 2.2 CDAI

The CDAI was derived from the UK Biobank baseline 24-h dietary recall data (collected via the Oxford WebQ questionnaire). The Dietary Antioxidant Index (DAI) for each nutrient was calculated based on the UK food composition database. The computational procedure was as follows: for each individual nutrient (Vitamin C, Vitamin E, β-carotene, Selenium, and Zinc), its intake level was standardized by subtracting the sample mean and dividing by the standard deviation. The composite CDAI score was calculated according to the formula below, representing the sum of the standardized values for the five constituent nutrients:


CDAI=∑t=1n=5(Individual Intake−Mean)SD

### 2.3 Incidence and mortality of AA/AD

Aortic aneurysm was defined as a focal or diffuse, permanent dilation of the aorta exceeding 1.5 times the normal expected diameter. The diagnosis of aortic dissection was established by the identification of an intimal flap that partitions the aortic lumen into true and false channels on imaging. Both the incidence of disease and mortality were ascertained through linkage to hospital inpatient records and national death registries within the UK Biobank database.

### 2.4 Covariates

Covariates were selected based on established risk factors identified in previous etiological studies of AA/AD, as well as data availability in the UK Biobank. The included variables comprised potential confounders associated with AA/AD. These variables includes demographic characteristics: sex (male/female), ethnicity (White/non-White), age (years), annual income (<£18,000; £18,000–30,999; £31,000–51,999; £52,000–100,000; >£100,000), socioeconomic status (Townsend deprivation index, TDI), and educational attainment (college or not). Lifestyle factors: smoking status (never/past/current), alcohol intake (never/past/current), physical activity (<600/600–3,000 />3,000 MET-minutes/week), body mass index (BMI), and BMI category (normal/lean/overweight or obese). Clinical measures: hypertension (yes/no/unknown), diabetes (yes/no), and cardiovascular disease (yes/no). Variables with less than 5% missing data were handled by multiple imputation, while those with more than 5% missing data were excluded from the analysis.

### 2.5 Statistical analysis

Participants were categorized into quartiles (Q1–Q4) based on their CDAI score, with the lowest quartile (Q1) serving as the reference group. Univariate and multivariate logistic regression models were employed to assess the association between CDAI and the risk of AA/AD incidence, with results expressed as odds ratios (ORs) and 95% confidence intervals (CIs). Cox regression models were used to evaluate the associations between all-cause mortality of AA/AD and both CDAI and components, with results presented as hazard ratios (HRs) and 95% CIs. Kaplan–Meier curves were plotted to visualize the relationships between CDAI levels and risk of AA/AD mortality, with between-group differences assessed using the log-rank test. Based on previously acquired data on daily nutrient intake, the individual effects of different antioxidants on these mortality outcomes were compared. Three adjusted models were constructed: Model 1 was adjusted for age and sex; Model 2 was further adjusted for smoking status, alcohol consumption, BMI, and physical activity level; Model 3 included all previous variables plus hypertension, diabetes, hyperlipidemia, LDL-C, and HbA1c. To investigate dose–response relationships, RCS with 4–5 knots were applied to test for linear or non-linear associations. Subgroup analyses were performed stratified by sex, age, history of hypertension, and BMI to identify populations potentially more susceptible to the influence of CDAI. Furthermore, a mediation analysis was performed to explore the potential anti-inflammatory effect of CDAI by examining its effect through four inflammation-related biomarkers.

## 3 Results

### 3.1 Population characteristics

The baseline characteristics of the 172,450 included participants, stratified by quartiles of CDAI, are summarized in Table 1. Significant differences (*p* < 0.001) were observed across quartiles in sex, age, ethnicity, education level, income, BMI, physical activity, smoking status, CVD, TDI, and diabetes. Notably, participants who were female, without hypertension, and classified as having a healthy BMI tended to have higher CDAI scores.

**Table 1:** Baseline characteristics of participants with CDAI data.

Characteristics	Categories	Total (*n* = 172,450)	CDAI (Q1) (*n* = 43,113)	CDAI (Q2) (*n* = 43,113)	CDAI (Q3) (*n* = 43,113)	CDAI (Q4) (*n* = 43,111)	*p*
Age (mean ± SD)		56.3 ± 7.9	55.9 ± 8.1	56.4 ± 7.8	56.5 ± 7.7	56.7 ± 8.0	<0.0001
Sex (%)	Female	93,506 (54.22)	25,373 (58.85)	24,878 (57.71)	23,341 (54.12)	19,914 (46.22)	<0.0001
	Male	78,944 (45.78)	17,740 (41.15)	18,235 (42.29)	19,772 (45.88)	23,197 (53.80)	
BMI (mean ± SD)		27.2 ± 4.4	27.6 ± 4.5	27.3 ± 4.4	27.1 ± 4.3	27.0 ± 4.2	<0.0001
BMI category (%)	Health	63,284 (36.70)	14,467 (33.55)	15,982 (37.07)	16,578 (38.45)	16,257 (37.72)	<0.0001
	Lean	918 (0.53)	223 (0.52)	211 (0.49)	236 (0.55)	248 (0.58)	
	Overweight or obese	108,248 (62.77)	28,423 (65.93)	26,920 (62.44)	26,299 (60.99)	26,606 (61.71)	
Activity (%)	0–600	25,650 (14.88)	7,489 (17.38)	6,851 (15.89)	6,121 (14.20)	5,189 (12.04)	<0.0001
	600–3,000	91,835 (53.25)	22,325 (51.78)	23,089 (53.55)	23,517 (54.55)	22,904 (53.13)	
	Over 3,000	54,965 (31.87)	13,299 (30.84)	13,173 (30.56)	13,475 (31.26)	15,018 (34.84)	
TDI index (mean ± SD)		−2.06 ± 3.05	−1.85 ± 3.18	−2.14 ± 3.02	−2.17 ± 2.98	−2.05 ± 3.08	<0.0001
Ethnicity (%)	White	164,724 (95.52)	40,352 (93.60)	41,487 (96.20)	41,563 (96.38)	41,322 (95.84)	<0.0001
	Non-White	7,726 (4.48)	2,761 (6.40)	1,626 (3.77)	1,550 (3.60)	1,789 (4.15)	
Drinking (%)	Never	5,643 (3.27)	1,751 (4.06)	1,314 (3.05)	1,178 (2.73)	1,400 (3.25)	<0.0001
	Past	5,206 (3.02)	1,470 (3.41)	1,207 (2.80)	1,169 (2.71)	1,360 (3.16)	
	Current	161,601 (93.71)	39,892 (92.54)	40,592 (94.15)	40,766 (94.56)	40,351 (93.60)	
Smoking (%)	Never	98,158 (56.92)	23,699 (54.97)	24,638 (57.12)	25,081 (58.17)	24,740 (57.40)	<0.0001
	Past	60,587 (35.14)	14,937 (34.65)	15,187 (35.21)	15,215 (35.29)	15,248 (35.38)	
	Current	13,705 (7.95)	4,477 (10.38)	3,288 (7.62)	2,817 (6.53)	3,123 (7.24)	
Income (%)	<18,000	26,890 (15.59)	7,845 (18.20)	6,923 (16.06)	6,558 (15.22)	5,564 (12.91)	<0.0001
	18,000–30,999	39,457 (22.88)	11,234 (26.05)	10,156 (23.56)	9,687 (22.47)	8,380 (19.43)	
	31,000–51,999	47,285 (27.42)	12,890 (29.89)	12,045 (27.93)	11,678 (27.09)	10,672 (24.76)	
	52,000–100,000	41,203 (23.89)	8,956 (20.77)	10,234 (23.73)	10,987 (25.48)	11,026 (25.58)	
	>100,000	17,615 (10.22)	2,188 (5.07)	3,755 (8.71)	4,203 (9.75)	7,469 (17.33)	
Education (%)	College	73,689 (42.74)	15,096 (35.02)	18,490 (42.87)	19,891 (46.13)	20,212 (46.88)	<0.0001
	No college	98,761 (57.26)	28,017 (64.98)	24,623 (57.13)	23,222 (53.87)	22,899 (53.12)	
Diabetes (%)	No	164,819 (95.57)	41,002 (95.14)	41,372 (95.94)	41,449 (96.12)	40,996 (95.12)	<0.0001
	Yes	7,631 (4.43)	2,111 (4.90)	1,741 (4.04)	1,664 (3.86)	2,115 (4.91)	
CVD (%)	No	162,034 (93.96)	40,416 (93.75)	40,661 (94.31)	40,667 (94.32)	40,290 (93.49)	<0.0001
	Yes	10,416 (6.04)	2,697 (6.25)	2,452 (5.69)	2,446 (5.67)	2,821 (6.54)	
Hypertension (%)	No	95,163 (55.19)	24,070 (55.83)	23,860 (55.33)	23,779 (55.14)	23,454 (54.40)	0.001
	Yes	70,735 (41.02)	17,547 (40.70)	17,612 (40.85)	17,697 (41.06)	17,879 (41.48)	
	Unknown	6,552 (3.80)	1,496 (3.47)	1,641 (3.81)	1,637 (3.80)	1,778 (4.13)	
TG (mean ± SD)	mmol/mol	1.75 ± 1.02	1.84 ± 1.08	1.78 ± 1.01	1.73 ± 0.98	1.66 ± 1.01	<0.0001
LDL-C (mean ± SD)	mmol/mol	3.57 ± 0.92	3.66 ± 0.96	3.60 ± 0.91	3.55 ± 0.89	3.47 ± 0.93	<0.0001
HbA1c (mean ± SD)	mmol/mol	35.8 ± 6.2	36.4 ± 6.8	35.9 ± 6.1	35.7 ± 5.9	35.2 ± 6.1	<0.0001
CDAI score (mean ± SD)		−0.35 ± 3.0	−3.85 ± 1.4	−1.38 ± 0.65	0.68 ± 0.72	3.89 ± 1.9	<0.0001

Continuous data were presented as the mean and 95% confidence interval, category data were presented as the proportion and 95% confidence interval. BMI, body mass index; TDI, townsend deprivation index; CVD, cardiovascular disease; TG, triglycerides; LDL-C, low-density lipoprotein cholesterol; HbA1c, glycosylated hemoglobin.

### 3.2 Association between CDAI and the incidence of AA/AD

**Figure 2:**
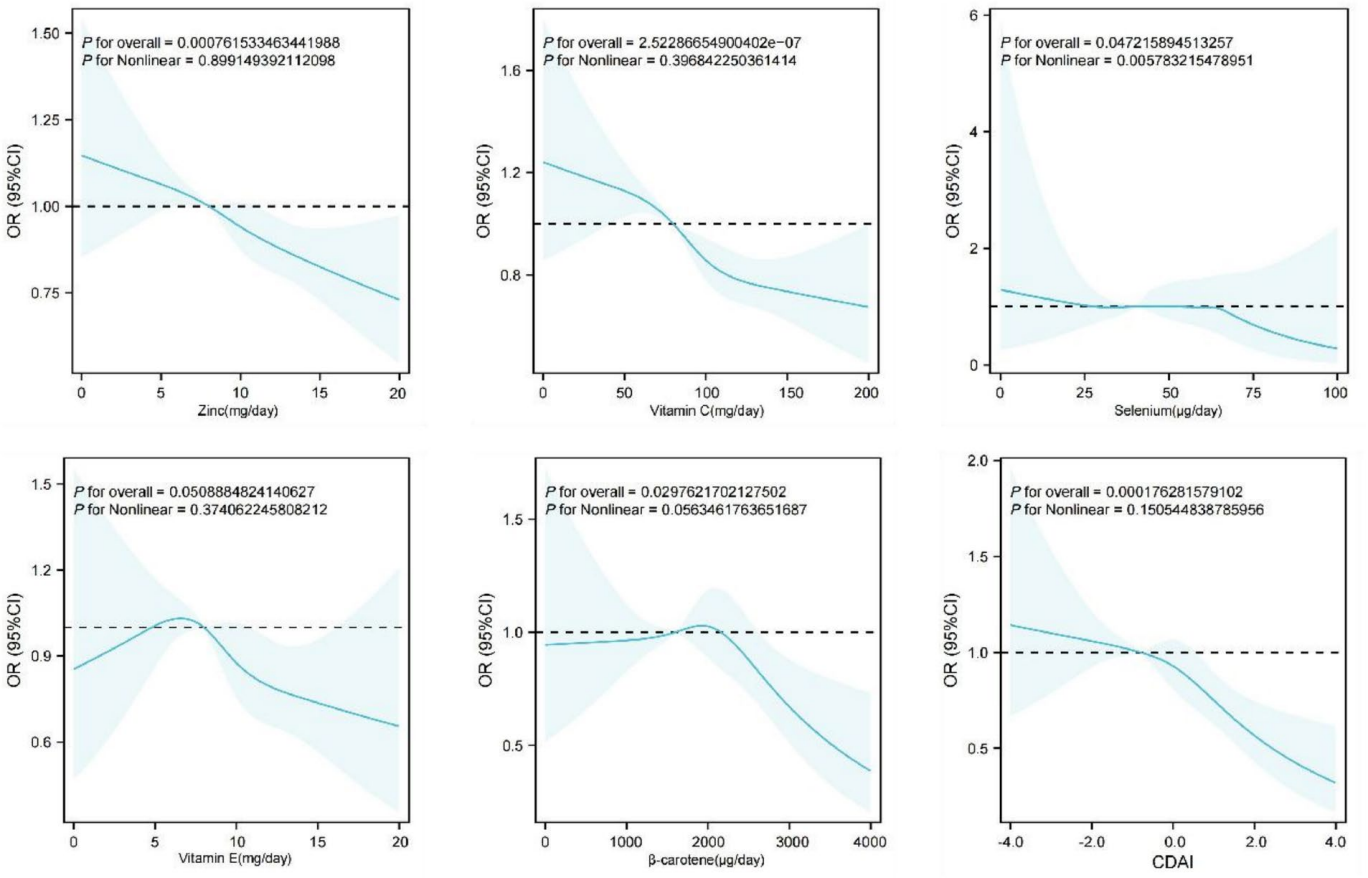
The correlation between each of zinc, vitamin C, selenium, vitamin E, β-carotene, CDAI, and the mortality of AA/AD.

We performed unweighted univariate and multivariate logistic regression analyses using the fully adjusted Model 3. As presented in Table 2, dynamic analysis integrating both univariate and multivariate results revealed that CVD, male sex (compared to female), education level, smoking, overweight, hypertension, and age were risk factors for AA/AD occurrence (OR >1, *p* < 0.001). Notably, CVD demonstrated a strong association with AA/AD development, remaining a significant risk factor even after controlling for confounders (OR = 2.30; 95% CI: 2.03–2.60). Conversely, increasing income and physical activity level showed gradually significant protective effects against AA/AD. Furthermore, higher CDAI values were inversely associated with AA/AD risk (OR = 0.93; 95% CI: 0.88–0.99). To evaluate the potential nonlinear relationships, restricted cubic splines were applied to model the associations of both the composite CDAI and its five constituent antioxidants with AA/AD. The results revealed linearly inverse associations of CDAI, zinc, vitamin C and *β*-carotene with the mortality of AA/AD, as presented in Figure 2.

**Table 2:** Univariate and multivariate logistic regression analysis for AA/AD risk factors.

Variables	Categories	OR (Univariate)	OR (Multivariate)
CVD	No	Ref	Ref
	Yes	3.87 (3.45–4.34), *p* < 0.001	2.30 (2.03–2.60), *p* < 0.001
Sex	Female	Ref	Ref
	Male	1.28 (1.07–1.53), *p* = 0.007	1.22 (1.01–1.47), *p* = 0.041
Income	Lowest income	Ref	Ref
	Low income	0.66 (0.58–0.74), *p* < 0.001	0.80 (0.70–0.90), *p* < 0.001
	Medium income	0.51 (0.45–0.58), *p* < 0.001	0.73 (0.64–0.84), *p* < 0.001
	Medium-high income	0.44 (0.38–0.50), *p* < 0.001	0.72 (0.62–0.83), *p* < 0.001
	High income	0.35 (0.28–0.43), *p* < 0.001	0.60 (0.47–0.76), *p* < 0.001
Education	High school or below	Ref	Ref
	College or above	1.51 (1.38–1.66), *p* < 0.001	1.17 (1.06–1.28), *p* = 0.002
Ethnicity	Primary ethnicity	Ref	Ref
	Other ethnicity	2.32 (1.99–2.69), *p* < 0.001	2.35 (1.99–2.78), *p* < 0.001
Drinking	No	Ref	Ref
	Yes	1.09 (0.85–1.42), *p* = 0.493	0.99 (0.75–1.30), *p* = 0.915
	Almost not	0.52 (0.43–0.63), *p* < 0.001	0.63 (0.51–0.77), *p* < 0.001
Smoking	Never	Ref	Ref
	Current	1.61 (1.48–1.76), *p* < 0.001	1.26 (1.15–1.38), *p* < 0.001
	Former smoker	1.24 (1.06–1.46), *p* = 0.009	0.98 (0.83–1.16), *p* = 0.832
Physical activity	Low activity	Ref	Ref
	Medium activity	0.63 (0.57–0.70), *p* < 0.001	0.70 (0.63–0.78), *p* < 0.001
	High activity	0.58 (0.52–0.66), *p* < 0.001	0.61 (0.54–0.69), *p* < 0.001
BMI	Underweight	Ref	Ref
	Normal weight	0.21 (0.03–1.47), *p* = 0.115	0.20 (0.03–1.42), *p* = 0.108
	Overweight	3.79 (3.35–4.28), *p* < 0.001	2.87 (2.53–3.26), *p* < 0.001
Hypertension	No	Ref	Ref
	Yes	1.52 (1.40–1.66), *p* < 0.001	1.11 (1.01–1.21), *p* = 0.031
	Unknown	1.01 (0.79–1.29), *p* = 0.950	1.02 (0.79–1.31), *p* = 0.874
Age	Per year	1.05 (1.04–1.06), *p* < 0.001	1.03 (1.03–1.04), *p* < 0.001
CDAI	Per unit	0.93 (0.88–0.99), *p* = 0.024	0.97 (0.92–1.03), *p* = 0.362
Social score	Per unit	1.07 (1.05–1.08), *p* < 0.001	1.05 (1.03–1.06), *p* < 0.001

Data are presented as OR (95% CI). CVD, cardiovascular disease; BMI, body mass index; CDAI, composite dietary antioxidant index. Adjusted variables: Age, sex, BMI, smoking status, alcohol consumption, physical activity, race/ethnicity, hypertension, coronary heart disease, TDI.

### 3.3 Association between CDAI and the mortality of AA/AD

**Figure 3:**
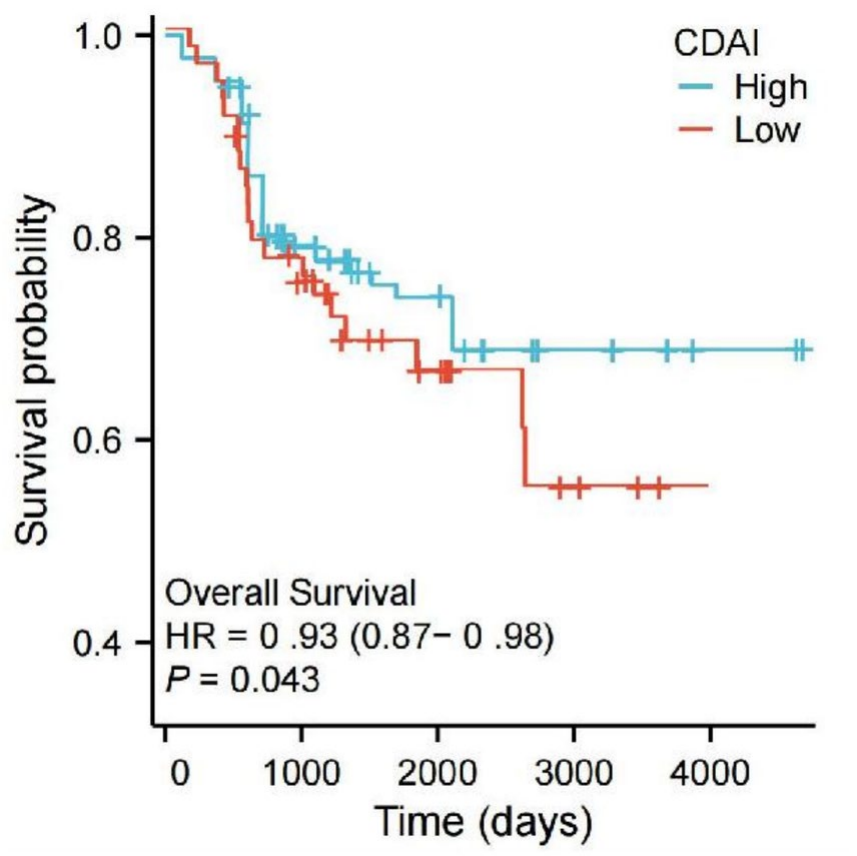
Kaplan–Meier survival curve.

Among the 172,450 eligible participants, 1,486 were diagnosed with AA/AD. Cox regression analyses demonstrated a significant inverse trend between CDAI levels and AA/AD mortality (P-trend < 0.001), which remained consistent after adjusting for covariates (*p* = 0.017), as presented in Table 3. Kaplan–Meier survival curves for AA/AD mortality across CDAI quartiles are shown in Figure 3. The curve for the lowest CDAI quartile declined more rapidly than that of the highest quartile, indicating a higher mortality rate. A significant survival difference was observed between the groups (HR = 0.93, 95% CI: 0.87–0.98, *p* = 0.043). We further evaluated the associations between individual nutritional components of CDAI and all-cause mortality from AA/AD. After full adjustment (Model 3), vitamin E and *β*-carotene showed significant inverse associations with AA/AD mortality (*p* < 0.05), as presented in Table 4.

**Table 3:** Association between baseline CDAI and the risk of AA/AD (*n* = 1,486).

Variables	HR1 (95% CI)	*p*	HR2 (95% CI)	*p*	HR3 (95% CI)	*p*
CDAI						
Q1	Ref		Ref		Ref	
Q2	0.89 (0.77–1.03)	0.115	0.93 (0.80–1.07)	0.298	0.94 (0.81–1.09)	0.405
Q3	0.81 (0.70–0.94)	0.006	0.86 (0.74–0.99)	0.045	0.88 (0.76–1.02)	0.095
Q4	0.72 (0.62–0.84)	<0.001	0.80 (0.69–0.93)	0.004	0.83 (0.71–0.96)	0.018
Per 1 increase	0.91 (0.87–0.95)	<0.001	0.94 (0.90–0.98)	0.015	0.95 (0.91–0.99)	0.043
P for trend		<0.001		0.004		0.017

Data are presented as HR (95% CI). HR1 adjusted for age, sex and ethnicity. HR2 adjusted for age, sex, ethnicity, assessment center, household income, education, TDI index, drinking status, smoking status, physical activity, body mass index. HR3 adjusted for age, sex, ethnicity, assessment center, household income, education, TDI index, drinking status, smoking status, physical activity, body mass index, diabetes, cardiovascular disease and hypertension.

**Table 4:** Association of five components of dietary antioxidants and all-cause mortality.

Components	Model 1		Model 2		Model 3	
	HR (95% CI)	*p*	HR (95% CI)	*p*	HR (95% CI)	*p*
Vitamin C	0.92 (0.85, 0.99)	0.031	0.94 (0.87, 1.02)	0.108	0.95 (0.88, 1.04)	0.276
Vitamin E	0.89 (0.82, 0.96)	0.005	0.91 (0.84, 0.98)	0.018	0.93 (0.85, 0.99)	0.048
β-carotene	0.86 (0.78, 0.94)	0.001	0.88 (0.81, 0.96)	0.006	0.90 (0.82, 0.98)	0.019
Selenium	0.85 (0.74, 0.97)	0.021	0.87 (0.76, 0.99)	0.042	0.89 (0.78, 1.02)	0.086
Zinc	0.87 (0.79, 0.96)	0.007	0.89 (0.81, 0.98)	0.023	0.91 (0.83, 1.00)	0.052

Data are presented as HR (95% CI). Model 1 adjusted for age, sex and ethnicity. Model 2 adjusted for age, sex, ethnicity, assessment center, household income, education, TDI index, drinking status, smoking status, physical activity, body mass index. Model 3 adjusted for age, sex, ethnicity, assessment center, household income, education, TDI index, drinking status, smoking status, physical activity, body mass index, diabetes, cardiovascular disease and hypertension.

### 3.4 Subgroup analysis

We performed subgroup analyses based on gender, age, hypertension, and BMI to explore the potential relationship between CDAI and AA/AD mortality across different strata, as shown in Table 5. When stratified by age, the highest quartile of CDAI was inversely associated with AA/AD risk among participants over 60 years old (HR = 0.82, 95% CI: 0.69–0.97), indicating an 18% reduction in mortality risk in Q4 compared to Q1. However, significant association of CDAI with mortality was not evident in the other subgroups.

**Table 5:** Subgroup analyses of association between baseline CDAI and the risk of AA/AD mortality.

Subgroup	Q1 (Ref)	Q2 HR (95% CI)	*p*	Q3 HR (95% CI)	*p*	Q4 HR (95% CI)	*p*	P for trend
Sex								
Male	1	1.02 (0.87–1.21)	0.811	1.05 (0.89–1.23)	0.568	0.97 (0.83–1.14)	0.721	0.382
Female	1	0.83 (0.63–1.09)	0.184	0.82 (0.61–1.10)	0.189	0.76 (0.56–1.02)	0.071	0.041
Age								
<60 years	1	1.08 (0.85–1.40)	0.521	1.18 (0.92–1.52)	0.194	1.17 (0.91–1.50)	0.223	0.156
≥60 years	1	0.94 (0.79–1.12)	0.487	0.92 (0.78–1.09)	0.341	0.82 (0.69–0.97)	0.019	0.021
Hypertension								
Yes	1	1.01 (0.83–1.22)	0.943	1.02 (0.84–1.24)	0.856	0.94 (0.78–1.15)	0.576	0.245
No	1	0.96 (0.78–1.18)	0.689	0.94 (0.76–1.16)	0.572	0.91 (0.74–1.12)	0.378	0.089
BMI								
Underweight	1	0.89 (0.58–1.36)	0.587	0.94 (0.62–1.43)	0.778	0.81 (0.53–1.24)	0.333	0.267
Healthy	1	0.97 (0.72–1.30)	0.836	0.99 (0.74–1.33)	0.951	0.93 (0.69–1.25)	0.628	0.134
Overweight/Obese	1	0.99 (0.85–1.16)	0.913	1.01 (0.86–1.19)	0.901	0.93 (0.79–1.09)	0.372	0.078

Data are presented as HR (95% CI). BMI, body mass index.

### 3.5 Mediation analysis

**Figure 4:**
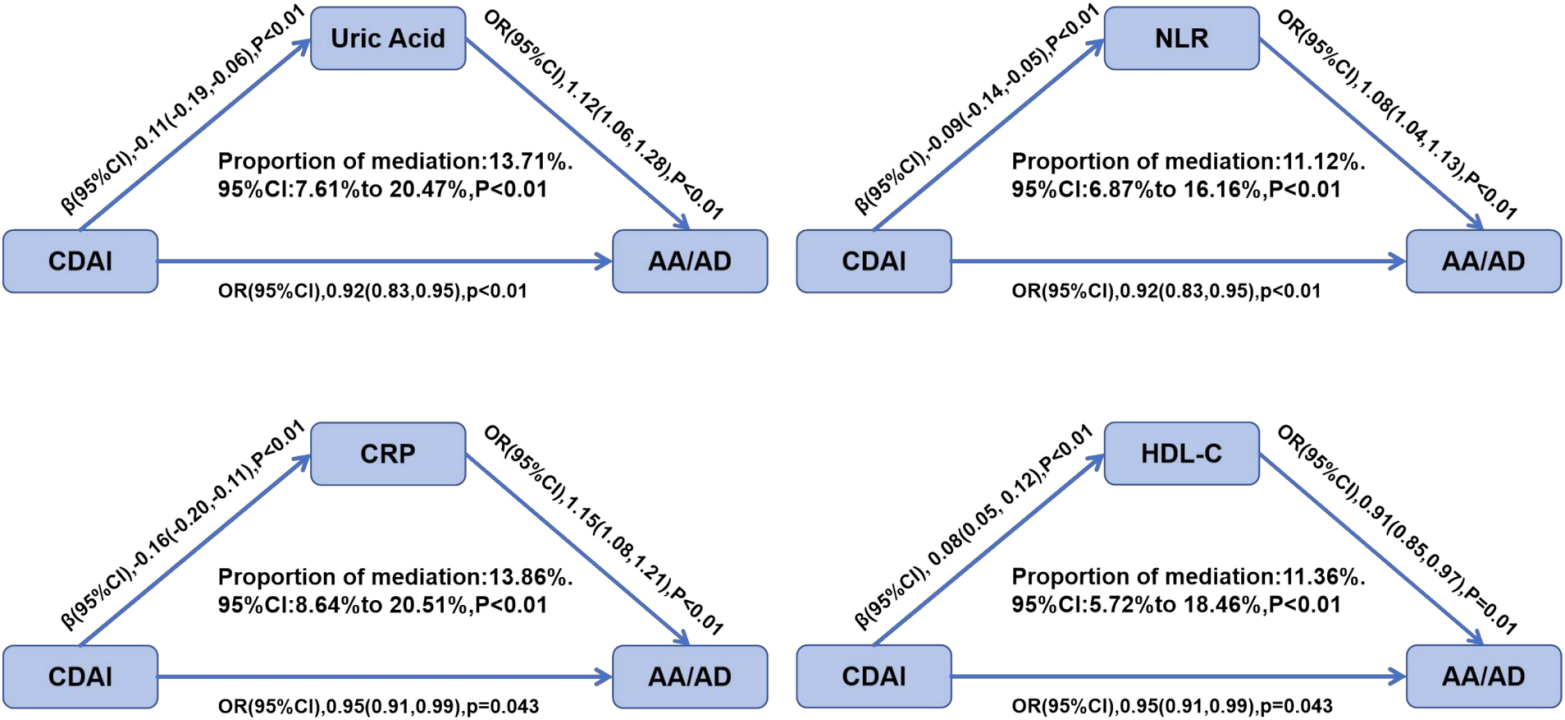
Mediation effects of uric acid, NLR, CRP, HDL-C on the associations of CDAI and AA/AD.

Mediation analysis indicated that metabolic indicators and inflammatory factors play mediating roles in the relationship between CDAI and AA/AD. As shown in Figure 4, uric acid, NLR, CRP, and HDL-C all demonstrated significant mediating effects (*p* ≤ 0.01). Specifically, uric acid, NLR, and CRP were inversely associated with CDAI (*β* < 0) but positively associated with AA/AD (OR > 1), mediating 13.71, 11.12, and 13.86% of the association between CDAI and AA/AD, respectively. In contrast, HDL-C was positively correlated with CDAI (*β* > 0) and inversely correlated with AA/AD (OR < 1), accounting for 11.36% of the mediation effect.

## 4 Discussion

Based on data from 502,250 participants in the UK Biobank database, we found that higher CDAI scores were inversely associated with AA/AD incidence in logistic regression models and with reduced mortality in Cox regression analyses. Additionally, RCS curves indicated linear inverse associations between AA/AD mortality and the intake of beta-carotene, vitamin C, zinc, and selenium, with significant protective effects observed for vitamin C and selenium.

The pathogenesis of aortic aneurysm and dissection involves complex mechanisms, including hemodynamic alterations, oxidative stress, inflammation, and extracellular matrix (ECM) remodeling, with the ultimate outcome likely resulting from interactions among these factors. Oxidative stress derives predominantly from elevated levels of ROS, including O₂^−^, H₂O₂, and −OH. Under physiological conditions, however, ROS serve beneficial effects as secondary messengers involved in signal transduction, immune defense, and the modulation of gene expression. When ROS production exceeds the detoxification capacity of cellular antioxidant systems, their strong oxidative potential induces protein and lipid peroxidation, as well as DNA damage (9). In the aorta, the detrimental effects of ROS are mediated primarily through the degradation of the ECM and apoptosis of vascular smooth muscle cells (VSMCs) (10). It has been demonstrated in animal studies that modulation of oxidative stress through inhibition of key antioxidant enzymes can influence the development and progression of aortic aneurysms. For instance, deficiency of Acetaldehyde Dehydrogenase 2(ALDH2) was associated with a higher incidence of abdominal aortic aneurysm in mice (11). Conversely, enhanced expression of the endogenous antioxidant enzyme superoxide dismutase 2 (SOD2) attenuates both aneurysm expansion and rupture (12). In the pathogenesis of AA/AD, inflammation and oxidative stress frequently act in synergy. Interleukin-6 (IL-6), a key inflammatory cytokine associated with various cardiovascular diseases, serves as a major biomarker of abdominal aortic aneurysm (AAA) and also is a key contributor to the formation of thoracic aortic aneurysms‌(TAA) (13). Furthermore, it can upregulate the expression of angiotensin II receptors and augment angiotensin II-induced superoxide production, thereby exacerbating oxidative stress (14). In patients with aortic dissection, the antioxidant defense system is often compromised and unable to effectively eliminate excessive ROS. Oxidative stress and inflammatory responses constitute a pathological nexus; each fuels the other in a self-sustaining cycle that results in the eventual breakdown of the aortic wall’s structural integrity. To investigate whether CDAI reduces the incidence of AA/AD by mitigating inflammation, mediation analysis was performed on common inflammatory markers and metabolic indicators, including uric acid, NLR, CRP, and HDL-C, with results indicating statistical significance.

Vitamins, as essential exogenous antioxidants, constitute a significant component of the CDAI score and primarily exert antioxidant effects by scavenging free radicals in the body (15). Among these, beta-carotene, a precursor of vitamin A, along with vitamin C, have been widely investigated for their effects on cardiovascular diseases. Some researchers hypothesize that these discrepancies may be attributed to geographical variations, dietary habits, ethnic differences, and adjustments for distinct confounding factors in statistical analyses (16). Moreover, synergistic interactions among multiple antioxidant nutrients may also contribute to the divergent findings reported in the literature. Such potential synergy underscores the rationale for employing a composite index. In our analysis of AA/AD mortality, each nutritional component was evaluated individually. The results revealed that both beta-carotene and vitamin C demonstrated a significant association with reduced AA/AD mortality. Similarly, analyses of the UK Biobank cohort have documented that higher dietary intake of vitamins C and E is associated with lower AA/AD incidence (17).

Selenium is an essential trace element for human health, involved in antioxidant activity, cancer prevention, maintenance of thyroid function, and cardiovascular disease protection (18). Of these roles, its antioxidant capacity and cardiovascular protective effects are the primary focus of this study. Selenium exerts its biological functions mainly through selenoproteins (19), notably glutathione peroxidase (GPx) and thioredoxin reductase (TrxR), in which Selenium serves as a central component that facilitates the clearance of peroxides and oxygen-free radicals (20). Selenium also modulates immune cell function and influences the production of inflammatory cytokines (21). Evidence indicates that selenium deficiency promotes a pro-inflammatory state characterized by elevated levels of cytokines including TNF-α, IL-1β, and IL-6, and reduced concentrations of anti-inflammatory mediators such as IL-10 (22). This pro-inflammatory state is closely linked to vascular inflammation and remodeling. Moderate selenium supplementation has been shown to restore this imbalance, reducing pro-inflammatory factor production and enhancing anti-inflammatory responses. Epidemiological evidence has linked low selenium status to an increased risk of incidence and mortality from cardiovascular diseases, suggesting that selenium may play a role in maintaining vascular health. Evidence from earlier investigations indicates a significant correlation between selenium levels and abdominal aortic aneurysm (AAA). Selenium deficiency has been shown to induce abnormal proliferation of aortic smooth muscle cells, leading to aortic expansion (23). Additionally, Witkowska et al. (24) found an inverse correlation between selenium levels and aneurysm diameter (*R* = –0.382), and lower dietary selenium intake has been documented in patients with thoracic aortic aneurysm compared to healthy individuals (25). Nevertheless, clinical studies directly examining the relationship between selenium and aortic dissection remain scarce, highlighting an important gap and a promising direction for future research.

Interestingly, synergistic interactions exist among different antioxidants. For instance, Burk et al. (26) reported that thioredoxin reductase, a selenoprotein, can reduce dehydroascorbic acid to ascorbic acid, indicating potential synergistic effects between antioxidants. However, the therapeutic window is crucial; administration of single antioxidants at supra-physiological doses, as documented for selenium, may induce paradoxical pro-oxidant activity. This suggests the existence of an optimal range for dietary antioxidant intake, which motivated our stratification of participants into groups based on CDAI levels. However, it should be emphasized that more antioxidants are not necessarily better. Supra-physiological doses of a single antioxidant may exert pro-oxidant effects, as exemplified by selenium in previous discussions. This implies the potential existence of an optimal range for dietary antioxidant intake. It was for this reason that we stratified the collected data into groups based on CDAI values in descending order. In baseline characteristics, certain health factors—such as normal BMI, never smoking, and no diabetes—were more prevalent in the Q3 group than in the Q4 group. However, in the analysis of AA/AD mortality, the Q4 group exhibited the highest hazard HR. This discrepancy may be attributed to population-specific effects, implying that CDAI influences the incidence and mortality of AA/AD to differing extents. Therefore, the optimal CDAI range warrants further investigation. In the baseline data, the proportion of favorable health profiles—such as normal BMI, never smoking, and absence of diabetes—was higher in the Q3 group compared to the Q4 group. However, analysis of AA/AD mortality revealed that the Q4 group had the highest HR. This discrepancy may be attributed to the distinct populations under investigation, suggesting that CDAI may differentially influence the incidence and mortality of AA/AD. Consequently, the optimal CDAI range remains to be determined through further research. Research has extensively examined the relationships of both the CDAI and specific antioxidants with cardiovascular diseases.

Supporting evidence comes from a recent investigation showing that subjects in the highest CDAI quartile exhibited reduced vascular age, indicating a positive correlation between elevated CDAI scores and improved aortic function (27). Another investigation revealed a nonlinear inverse association between CDAI and coronary heart disease among US adults (28). More directly, serum levels of vitamin C, beta-carotene, zinc, and selenium were shown to be significantly inversely correlated with increasing AAA diameter (29). Furthermore, CDAI has also demonstrated protective effects against other diseases, such as lung cancer and Cardiovascular-kidney-metabolic syndrome (30, 31). These conditions share common risk factors with AA/AD, indirectly supporting the potential protective role of CDAI against AA/AD. Since different antioxidants are derived from various food sources, a comprehensive dietary pattern rich in grains, vegetables, fruits, and nuts may facilitate the intake of natural antioxidants. This has practical clinical implications: individuals with risk factors such as male sex, hypertension and advanced age may reduce their risk of developing AA/AD by adopting dietary modifications aimed at increasing natural antioxidant consumption. In the baseline data, Our analysis revealed an inverse relationship between CDAI levels and the prevalence of cardiometabolic risk factors, including diabetes, elevated LDL-C, higher HbA1c, and hypertriglyceridemia. Conversely, higher CDAI was correlated with increased proportions of individuals engaging in substantial physical activity (≥3,000 MET-min/week), having a normal BMI, and being non-smokers. This suggests that an antioxidant-rich diet may exert its maximal protective effect against AA/AD when combined with other healthy lifestyle behaviors—such as regular physical activity, smoking avoidance, and maintenance of a healthy body weight.

This study has several notable strengths. Firstly, our study represents the first investigation to delineate the role of CDAI in the development of AA/AD, thereby offering novel perspectives on dietary interventions aimed at preventing both the incidence and mortality of these conditions. Furthermore, the findings provide substantive evidence supporting the involvement of inflammation and oxidative stress in the pathogenesis of AA/AD. This study also has several limitations. First, the UK Biobank database does not include information on the Stanford classification of aortic dissections. Since type A dissections, which involve the ascending aorta, are associated with higher mortality than type B dissections (32), our results cannot elucidate the potential differential effects of CDAI across dissection subtypes. Second, as the UK Biobank draws predominantly from a British cohort, caution should be exercised when extrapolating these findings to populations of different ethnic backgrounds and geographic locations. Third, the CDAI was calculated based on participants’ recall of their dietary intake over the previous 24 h, which is susceptible to measurement error. Fourth, although the CDAI incorporates major dietary antioxidants, other antioxidant compounds—such as polyphony and polyacrylamide—were not included. These unmeasured antioxidants may influence the outcomes and represent a source of unaccounted confounding, Finally, due to data availability constraints in the UK Biobank, we were unable to investigate the mediating effects of key cytokines such as IL-1β, IL-6, and TNF-α.

## 5 Conclusion

This study demonstrates a significant inverse association between CDAI and both the incidence and mortality of AA/AD. The findings provide novel insights into dietary intervention strategies for the prevention of AA/AD and offer practical nutritional guidance for high-risk populations, thereby underscoring the importance of CDAI in the context of aortic diseases.

## Data Availability

The original contributions presented in the study are included in the article/supplementary material, further inquiries can be directed to the corresponding authors.
